# Prediction of Mental Health Problems Among Children Using Machine Learning Techniques

**DOI:** 10.1192/j.eurpsy.2025.841

**Published:** 2025-08-26

**Authors:** A. Leonova, K. Vasilchenko, T. Raeva

**Affiliations:** 1 Pain Treatment Clinic, Tyumen, Russian Federation; 2 The Human artificial control Keren (HacK) lab, Azrieli Faculty of Medicine, Bar-Ilan University, Safed, Israel; 3 Psychiatry and Addiction Medicine, Tyumen State Medical University, Tyumen, Russian Federation

## Abstract

**Introduction:**

Developmental language delay in children is characterized by the qualitative and quantitative underdevelopment of vocabulary and insufficient expressive language skills. These delays frequently co-occur with mental health issues including the emotional and behavioral sphere difficulties and it may result in adaptation difficulties in school. A multifactorial etiology has been identified for developmental language delay, highlighting the necessity for early prediction and intervention tools. The early prediction of speech and language delays in children under one year of age can help to prevent complications of the existing impairments in future.

**Objectives:**

To develop an algorithm of developmental language delay prediction in toddlers, based on anamnesis data.

**Methods:**

The training dataset had been collected from anamnesis of 232 children aged 18 till 36 months. Signs of developmental language delay had been presented in 50% children; typical language development had been presented in the other half of the sample. The neural network architecture had been developed using the Python 3.0 programming language and the Keras library. The compiled neural network had been trained on data which represented anamnesis of children under 1 year old. 70% of the collected data array were used for neural network training, 30% were used for validation.

**Results:**

The algorithm architecture consisted of direct propagation neural network of 5 Dense layers. A one-dimensional tensor of 58 values had been fed to the input of the network. At the output, the value of the probability of developmental language delay had been obtained. During 1000 of training iterations the accuracy of 95% had been achieved. Sensitivity and specificity of the model reached 95% and 100%, respectively.

**Conclusions:**

For the first time in the world, a neural network model of predicting the developmental language delay in early age children had been developed. This model considers data of intrauterine period of development, as well as the life of a child up to 1 year. It may help to prevent severe mental disorders which are comorbid to speech disorders.

**Disclosure of Interest:**

None Declared

